# A Unique Case of Bilateral Valsalva Retinopathy

**DOI:** 10.18502/jovr.v14i4.5471

**Published:** 2019-10-24

**Authors:** V G Madanagopalan, Girish Velis

**Affiliations:** ^1^Vitreo-Retinal Services, Aravind Eye Hospital, Thavalakuppam, Pondicherry, India

##  PRESENTATION

**Figure 1:**
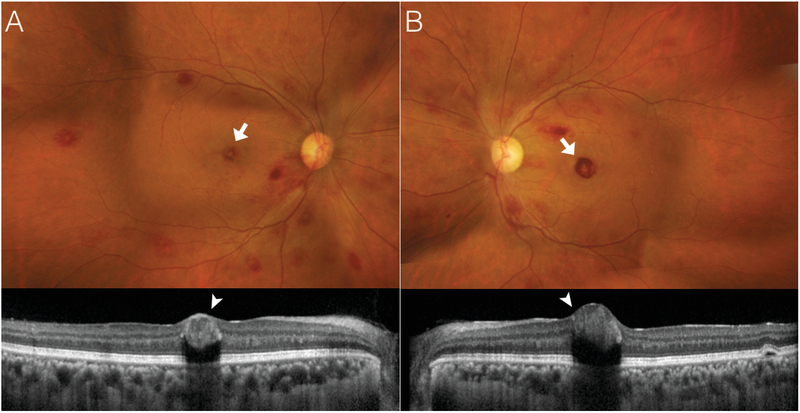
A montage of images of the retina and macular optical coherence tomography (OCT) in the right eye (Panel A), and left eye (Panel B). Multiple superficial retinal hemorrhages are seen in all quadrants. The hemorrhages at the fovea (arrows) are responsible for defective central vision. The presence of hemorrhages obliterating the foveal photoreceptors is confirmed on OCT (arrowheads).

A 45-year-old man presented to the eye clinic with complaints of diminished bilateral central vision for four days. He had constipation and bleeding during defecation over the past five months. He experienced severe straining accompanied by a particularly heavy episode of bleeding that occurred five days ago. Thereafter, a surgeon diagnosed an anal fissure that was treated with 2% topical glyceryl trinitrate ointment. Visual acuity in both eyes was 20/200, and the examination of the anterior segment was normal. Retinal examination with an indirect ophthalmoscope revealed multiple hemorrhages in all quadrants of the retina (Figures 1(A), right eye; 1(B), left eye). The fovea in both eyes was obscured by hemorrhages that was responsible for the mechanical obstruction of the central vision (arrows). Hemorrhages were noted at the center of the macula as well (arrows). These hemorrhages beneath the internal limiting membrane (ILM) were confirmed with optical coherence tomography (OCT), obliterating the foveal dip (arrowheads). A diagnosis of Valsalva retinopathy was made. Hematological investigations showed a hemoglobin level of 7 g/dl (normal range: 13.5 to 17.5 g/dl) and a platelet count of 140,000 (normal range: 150,000 to 450,000). The patient was asked to continue the treatment with his surgeon and was reviewed regularly at the eye clinic. The treatment for the anal fissure was successful, and he was relieved of constipation in one month. Three months later, visual acuity in both eyes improved to 20/20. Retinal examination showed a complete resolution of hemorrhages. Hematological evaluation revealed that the hemoglobin level was 13.8 g/dl and the platelet count was 270,000.

##  Discussion

Valsalva retinopathy was described by Duane in 1972.^[1]^ In contrast to both retinal arterial and venous systems being affected in Purtscher's retinopathy, the preretinal hemorrhages noticed in Valsalva retinopathy are attributed to the changes in the venous system alone. An abrupt increase in intra-thoracic or intra-abdominal pressure– particularly against a closed glottis as may occur with coughing, vomiting, lifting weights, or straining – causes visual loss due to premacular hemorrhages.^[2]^ In Valsalva retinopathy, the premacular hemorrhage can be a subhyaloid hemorrhage or a sub-ILM hemorrhage.^[3]^ In our patient, fissure in ano was the cause of constipation. Consequently, straining during stools was responsible for Valsalva retinopathy. With the hematological profile presented, the possible role of anemia could also be considered in this patient. Anemic retinopathy presents with extravasation of blood into the retina due to retinal vasodilation as a response to relative retinal ischemia in the setting of acute blood loss.^[4]^ As the sub-ILM hemorrhage at the fovea in both eyes was small in size, the patient was not subjected to ocular treatment. While encountering larger premacular hemorrhages, clinicians may utilize laser hyaloidotomy or vitrectomy to evacuate the hemorrhage.^[3,5]^

The images presented demonstrated the retinal changes responsible for visual loss in both eyes, encountered by a patient who developed Valsalva retinopathy secondary to anal fissure.

##  Financial Support and Sponsorship

Nil.

##  Conflicts of Interest

There is no conflict of interest.
